# Construction of a prognostic prediction model in liver cancer based on genes involved in integrin cell surface interactions pathway by multi-omics screening

**DOI:** 10.3389/fcell.2024.1237445

**Published:** 2024-02-05

**Authors:** Xiang Yu, Hao Zhang, Jinze Li, Lu Gu, Lei Cao, Jun Gong, Ping Xie, Jian Xu

**Affiliations:** ^1^ Department of Radiology, Sichuan Provincial People’s Hospital, University of Electronic Science and Technology of China, Chengdu, China; ^2^ Department of Radiology, Chinese Academy of Sciences Sichuan Translational Medicine Research Hospital, Chengdu, China; ^3^ Department of Hepatobiliary Surgery, Sichuan Provincial People’s Hospital, University of Electronic Science and Technology of China, Chengdu, China; ^4^ Department of Hepatobiliary Surgery, Chinese Academy of Sciences Sichuan Translational Medicine Research Hospital, Chengdu, China

**Keywords:** liver cancer, multi-omics data, integrin cell surface interactions, prognosis model, ITGAV

## Abstract

**Background:** Liver cancer is a common malignant tumor with an increasing incidence in recent years. We aimed to develop a model by integrating clinical information and multi-omics profiles of genes to predict survival of patients with liver cancer.

**Methods:** The multi-omics data were integrated to identify liver cancer survival-associated signal pathways. Then, a prognostic risk score model was established based on key genes in a specific pathway, followed by the analysis of the relationship between the risk score and clinical features as well as molecular and immunologic characterization of the key genes included in the prediction model. The function experiments were performed to further elucidate the undergoing molecular mechanism.

**Results:** Totally, 4 pathways associated with liver cancer patients’ survival were identified. In the pathway of integrin cell surface interactions, low expression of COMP and SPP1, and low CNVs level of COL4A2 and ITGAV were significantly related to prognosis. Based on above 4 genes, the risk score model for prognosis was established. Risk score, ITGAV and SPP1 were the most significantly positively related to activated dendritic cell. COL4A2 and COMP were the most significantly positively associated with Type 1 T helper cell and regulatory T cell, respectively. The nomogram (involved T stage and risk score) may better predict short-term survival. The cell assay showed that overexpression of ITGAV promoted tumorigenesis.

**Conclusion:** The risk score model constructed with four genes (COMP, SPP1, COL4A2, and ITGAV) may be used to predict survival in liver cancer patients.

## Introduction

Liver cancer is a common cancer and cause approximately one million deaths each year (Borlak et al., 2015). Early stage of liver cancer is usually asymptomatic, and the majority of patients are diagnosed at advanced stages. Furthermore, a significant proportion of liver cancer patients also suffer from liver fibrosis or cirrhosis (Farazi and DePinho, 2006). Prognosis for patients with advanced liver cancer is poor with lower overall survival rate (less than 5%). Although radical resection is the optimal treatment option for early-stage patients, the overall survival is still dismal (Hanazaki et al., 2000). The poor survival rate can be attributed to tumor relapse and inhomogeneity of primary tumors (Cha et al., 2002; Chen et al., 2017). In view of this, seeking for the molecular markers to predict the prognosis of liver cancer are urgently needed.

It is found that increased gene copy number variations (CNVs) contributed to the overexpression of granulin precursor (GEP) in a subset of liver cancer (Yung et al., 2015). Frequent mutations of tumor protein p53 (TP53) gene are detected in liver cancer (Zucman-Rossi, 2010). Aberrant methylation of dynein axonemal heavy chain 17 (DNAH17) is associated with comprehensive clinic-pathological factors and can serve as a potential biomarker for tumor thrombosis in liver cancer patients (Fan et al., 2019). In cancers, identification of survival-associated cellular processes will provide more information. Recently, multi-omics data (such as gene expression, CNVs, mutations, and DNA methylation) integration is a promising method to improve patients’ outcome (Werner et al., 2014; Rabbani et al., 2016; Lightbody et al., 2019). Proper processing and in-depth analysis of these multidimensional and diverse data makes it possible to obtain comprehensive and reliable insights. In this study, we used Multi-Omics Survival Clip (MOSClip) for the first time to integrate multi-omics data (gene expression, mutations, CNVs, and DNA methylation) of liver cancer to explore pathways or models associated with patient survival. We assessed the relationship between omics data with survival time, and further constructed a prognostic model for predicting survival rate in liver cancer patients.

## Materials and methods

### Data retrieval and cleaning

We used the TCGABiolinks package in R to retrieve expression, DNA methylation, CNVs and somatic cell mutation data in the TCGA database. All data were referred to the human HG38 genome. Only primary liver cancer tumor specimens from patients with survival data were selected for further analysis. The data format of “HTSeq-Counts” was selected to obtain the gene expression data from TCGA database. Only those genes with expression amount >100 in all samples were retained for subsequent machine learning analysis. The data were standardized and log_2_ converted through the EDAseq package in R. The DNA methylation data were downloaded from Illumina Human Methylation 450K platform. The R package methylMix (with default parameter) was used to group the CpG islands. Genes with no detection values in more than 60% of patients were excluded. The β value, i.e., the association between different sites and relevant gene promoter, was defined as the percentage of gene promoter methylation sites (Gevaert, 2015). The CNVs matrix was downloaded by using the getGistic function of type “thresholds” in the form of “genes per patient”. ±2, ±1 and 0 represented severe amplification/deletion, mild amplification/deletion and normal, respectively. The mutect2 pipeline was used to download somatic mutations data. The influence degree of mutations was defined according to the VEP software (McLaren et al., 2016). The data were converted to a Boolean sparse matrix of genes for each patient, indicating the presence or absence of mutations.

### Data dimension reduction

As serious redundant information exists in the omics data, dimensionality reduction of these data is required. MOSClip is a new tool for survival path analysis of multiple omics (Martini et al., 2019). Biomarkers affecting the patient survival were identified through the pathway and module analysis. In the MOSClip model, after dimensionality reduction, multiple omics data were taken as co-variables of Cox Proportional Hazards Regression Model, and the survival data of patients were taken as response variables for testing. Different dimensionality reduction methods are used for different omics data. Dimensionality reduction was performed for gene expression data by the principal component analysis (PCA). Hierarchical clustering was performed for DNA methylation data. For mutation data, binary counting method was used to reduce dimensionality. Downloaded CNVs data were already in integer format, no processing was done for the CNVs data.

### Pathway and module analysis

The pathway data were downloaded from the Reactome database and filtered by using gene pairs. For the pathway analysis, we screened 10 pathways with more than 10 genes. Then, resampling strategy (resampling success rate >80%) was adopted to select pathways. The Cox Proportional Hazards Regression model was applied for survival analysis. The maxClique function in RBGL of R package was used to identify the module. For module analysis, we only selected pathways involving 20–100 genes. After decomposing into many small modules, pathways were selected through the resampling strategy (resampling success rate >80%). For a given pathway or module, we implemented different strategies to identify the genes most relevant to survival. The absolute value of gene load and Kruskall-Wallis test was used in PCA and cluster analysis, respectively. For binary data, we only screened genes with mutation, increase, or decrease of the copy number.

### Construction of prognostic risk scoring model based on key genes in specific pathway

RNA sequencing data of key genes in specific pathway were downloaded from the UCSC Xena database (including 368 cases and 50 normal controls) to construct a prognostic risk scoring model. GSE141198 dataset (including 146 cases) and GSE144269 dataset (including 70 cases and 70 normal controls) were downloaded from the Gene Expression Omnibus (GEO) database. With the criteria that the dataset should contain information on patient survival, GSE141198 dataset was used to verify the prognosis model. GSE144269 dataset was used to verify the expression of key genes. Based on the expression of key genes in the specific pathway, variables were screened using the least absolute shrinkage and selection operator (LASSO) method. Multi-factor Cox regression model was used to construct the risk score model of related genes. The calculation formula is as following: Risk Scores = Σ Coef (i) * Exp (i). The median risk score was used as the cut-off point to divide patients into the high- and low-risk groups. The Kaplan-Meier and time-dependent ROC curve were used to analyze the survival curve and verify the accuracy of the risk score.

### Relationship between risk score and clinical features

Univariate and multivariate Cox analysis were used to determine whether the risk score was an independent prognostic factor. After testing collinearity, all independent prognostic parameters and related clinical parameters were included to construct a stepwise Cox regression model to predict 1-, 3- and 5-year overall survival of liver cancer patients. A nomogram was drawn to calibrate the curve to compare predicted and observed overall survival. The potential net benefit of nomogram and risk score with other independent prognostic factors was compared using decision curve analysis (DCA). In addition, differences of risk score among different clinical subgroups were compared.

### Molecular characterization of key genes in risk model

Firstly, the expression levels of key genes in the risk model were evaluated in TCGA database with Wilcox test method, and verified in the GSE144269 dataset. Secondly, UALCAN database was used to investigate the protein expression of key genes in liver cancer patients. Thirdly, the GSE36915 dataset (including 68 cases and 21 normal controls) was downloaded to identify differentially expressed microRNAs (miRNAs) with |log_2_ fold change (FC)| >0.6 and *p* < 0.05. ENCORI database was used to obtain the miRNAs targeted to key genes. Those differentially expressed miRNAs that negatively regulated key genes were intersected with the predicted results in the database to obtain the miRNA-mRNA pairs. In addition, ENCORI database was used to acquire long non-coding RNAs (lncRNAs) targeted by miRNAs obtained above, which were overlapped with lncRNAs that positively correlated with key genes (pearson correlation coefficient >0.2 and *p* < 0.05) to obtain the lncRNA-miRNA pairs, followed by network construction between lncRNAs, miRNAs and key genes. Fourthly, according to the median expression value of key genes in each sample, all samples were divided into the high- and low-expression groups. Kaplan-Meier analysis was used to evaluate the influence of key genes on survival. Fifthly, receiver operating characteristic (ROC) curve was utilized to estimate the potential diagnostic value of key genes using the pROC package. Area under the curve (AUC) is an evaluation index of model performance. Finally, those drugs associated with key genes were identified based on the DGIdb database.

### Analysis of tumor immune microenvironment (TIME) cell infiltration

The single sample gene set enrichment analysis (ssGSEA) algorithm was applied to quantify the relative abundance of immune cells in TIME of liver cancer. The enrichment score was used to represent the relative abundance of infiltrating cells in each sample to observe the difference of immune cell infiltration between high- and low-risk groups. The “ESTIMATE” in R was used to calculate the immune score, matrix score, tumor purity, and ESTIMATE score of each patient. In addition, correlation analysis was performed for key genes, risk score, and immune cells. Finally, the expression of some immune checkpoints in the high- and low-risk groups, and the association between immune checkpoints and risk score was evaluated and analyzed, respectively.

### Real time qPCR (RT-qPCR) validation of key genes associated with patient survival

To further investigate the expression patterns of key genes related to patient survival, the RT-qPCR was performed in the blood samples of 20 patients who relapsed after 3 and 6 months. The inclusion criteria were as follows: (1) Patients were diagnosed with liver cancer, which were confirmed by pathological examination; (2) Patients had not receive radiotherapy or chemotherapy prior to diagnosis; (3) Patients had no other malignant tumors. The exclusion criteria were as follows: (1) Patients complicated with other malignant tumors or viral infections; (2) Patients received adjuvant chemotherapy or targeted therapy before surgery; (3) Patients had incomplete clinical data. All individuals provided informed consent with the approval of the ethics committee of Sichuan Academy of Medical Sciences & Sichuan Provincial People’s Hospital (2020-101).

Total RNA of the blood samples was extracted using the RNAliquid hypervelocity whole blood (liquid sample) total RNA extraction kit (Beijing Huitian Oriental Technology Co. LTD). 1 μg RNA was applied to synthesize cDNA by FastQuant Reverse Transcriptase (TIANGEN). Then RT-qPCR was performed in an ABI 7300 Real-time PCR system with SYBR^®^ Green PCR Master Mix (Applied Biosystems). Relative gene expression was analyzed by fold change method.

### Cell culture and transfection

Based on the preliminary experimental results (data not shown), two cell lines (Hepg2 and Hep3B2-1-7) and one molecule (ITGAV) were selected for further molecular mechanism studies. Hepg2 and Hep3B2-1-7 cell was respectively used for overexpression and knockdown experiment, followed by cell proliferation, cell cycle, cell apoptosis, cell scratches and transwell assay, and Western blotting analysis. Hepg2 and Hep3B2-1-7 cell were cultured in Minimum Essential Medium (MEM) medium (Gibco, USA) containing 10% of fetal bovine serum, 1% of non-essential amino acid, 1% of double antibiotics, and 5% of CO_2_ at 37°C incubator. The transfection was performed according to Lipofectamine™ 2000 kit (Invitrogen, CA).

### Cell counting kit-8 (CCK8) assay

The cells were digested by trypsin (Gibco, USA), counted, and adjusted to concentration of 1 × 10^4^ cells/mL. 100 μL of cell suspension was added to each well of the 96-well plate and cultured for 4 h. According to the given concentration gradient of gatifloxacin, three compound wells were set for each gradient, and the corresponding concentration of gatifloxacin was added to each well. Subsequent tests were conducted after 24 h of culture. 10 μL of CCK-8 solution (APExBIO, United States) was added to the 96-well cell culture plate and incubated for 1 h. The absorbance at 450 nm was measured with a microplate reader (Bio-Rad, United States).

### Cell cycle analysis

Cell cycle agent (4A BIOTECH, Suzhou, China) was used to perform cell cycle analysis. 4 mL of 95% pre-cooled ethanol (Sinopharm Chemical Reagent Co., Ltd., Shanghai, China) was added into 1 ml of cell suspension drop by drop and fixed at 4°C for 2 h or longer. The cells were centrifuged at about 1,000 rpm for 3–5 min. 5 ml of pre-cooled PBS (MDL) was added into supernatant. The cells were re-suspended, and the cells were centrifuged again to precipitate. After removing the supernatant, the cells were dispersed appropriately. 0.4 mL of propidium iodide staining solution was added to each tube of the cell sample. Cell precipitation was slowly and fully suspended, and bathed at 37°C for 30 min in darkness for further cell cycle analysis by flow cytometry.

### Cell apoptosis analysis

The cell apoptosis was assessed using apoptosis agent (4A BIOTECH). The cells were digested with trypsin and incubated with medium. All adherent cells were blown off and gently blown away. The cells were collected and centrifuged at 1,000 rpm for 5 min. After removing the supernatant, 1 mL of pre-cooled PBS was added into cells for suspension. The cells were centrifuged again and aspirated the supernatant. The cells were suspended with binding buffer. 100 μL of cell suspension was mixed with 5 μL of Annexin V/FITC and incubated at room temperature under dark conditions for 5 min. Cells were mixed with 10 μL of propyl iodide solution and 400 μL of PBS for cell apoptosis analysis with a flow cytometer (BD, USA).

### Cell scratches and transwell assay

When the cells seeded in 6-well plate reached a confluent state, a single scratch was made using a sterile 10 μl pipette tip. The cells were then incubated with serum-free culture medium in 5% of CO_2_ incubator at 37°C. Images of the scratches were captured at 0, 24 and 48 h under a microscope (Novel, XS-2100). The width of the scratch was analyzed using ImageJ software according to the protocol (https://imagej.nih.gov/ij/docs/guide/index.html). The migration rate was calculated as follows: (scratch area at 0 h—scratch area at target time)/scratch area at 0 h *100%.

Before the experiment, the cells were starved in serum-free medium for 12 h. The cells were digested with trypsin and centrifuged at 1,000 rpm for 3 min. After removing the supernatant, the cells were washed with PBS. After supernatant was discarded, the cells were suspended in serum-free medium containing 0.1% of BSA for cell count. The cell density was adjusted to 1 × 10^5^/mL in serum-free medium containing 0.1% of BSA. 500 μL of complete medium was added into 24-well plate. 200 μL of cell suspension was added in transwell chamber. The cells were transferred to a 24-well plate containing the complete culture medium and incubated for 24 h in the cell incubator. After removing the upper chamber medium, the cells were wiped with the moistened cotton swab. The bottom of Transwell chamber was immersed in 10% of the methanol solution to fix the cells for 30 s and transferred to pure water. After washing off the methanol, the bottom of transwell chamber was immersed in crystal violet dye for dyeing for 2 min, and cleaned with pure water until the background was clear. Microscope photography was performed.

### Western blotting analysis

The protein was extracted from cells using RIPA kit (Beyotime, Shanghai, China). The quantification of the protein concentrations was analyzed using a BCA Protein Assay Kit (MDL, Beijing, China). Then, protein samples were separated via SDS-PAGE (Bio-Rad, United States) and transferred onto 0.22 μm polyvinylidene fluoride membrane (Millipore, Billerica, MA). The membrane was blocked with 5% non-fat dried milk for 1 h, incubated overnight with diluted anti-actin (1:1,000, T0022, CST), anti-Bcl-2 (1:2000, bs-0032R, BIOSS), anti-ITGAV (1:2000, bs-2203R, BIOSS), anti-MAPK (1:2000, bs-0637R, BIOSS), and anti-PXN (1:2000, YT-3606, Immuno Way) on a shaker at 4°C, and then rinsed in TBST for three times (10 min each time). Then, the membrane was incubated with HRP-conjugated affinipure goat anti-rabbit IgG (H + L) (Proteintech, China) for 1 h at room temperature, followed by a wash in TBST. The protein bands were visualized by using ECLTM Western blotting Detection Reagents using ChemiDoc MP Chemiluminescence imaging System (Bio-Rad, United States). The relative expression of protein was calculated as the ratio of the gray value of target protein to the gray value of the reference using ImageJ software according to the protocol (https://imagej.nih.gov/ij/docs/guide/index.html).

## Results

### Pathway and module analysis

**FIGURE 1:**
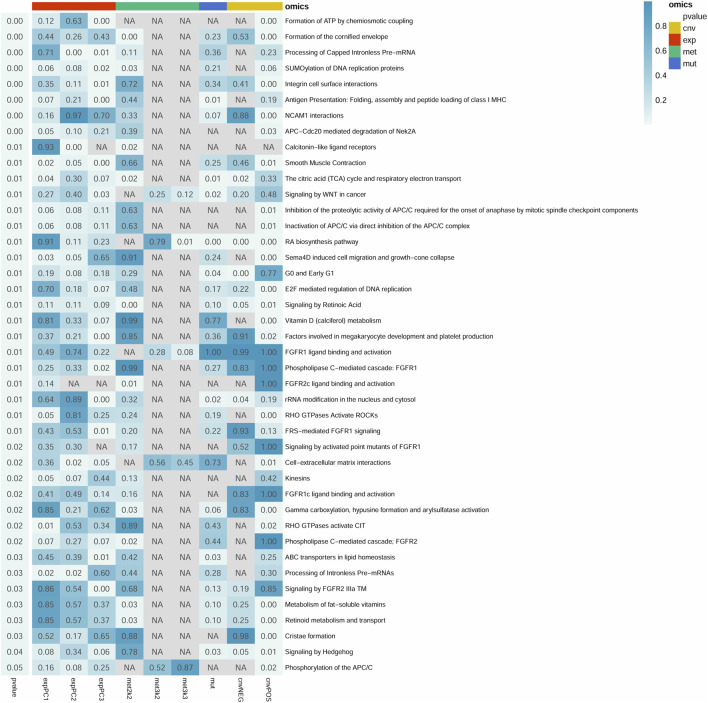
Summary of 42 significant pathways (*p*-value <0.05 and resampling >80%). Row and column represents pathway and omics, respectively. Right *p*-value represents the pathway significance in the Cox survival analysis. *p*-value in the box represents the significance of different omics in the pathway. NA represents no omics in the pathway. cnv: copy number variation data; exp: gene expression data; met: DNA methylation data; mut: mutations data. The cnvPOS and cnvNEG represents positive and negative, respectively.

**FIGURE 2:**
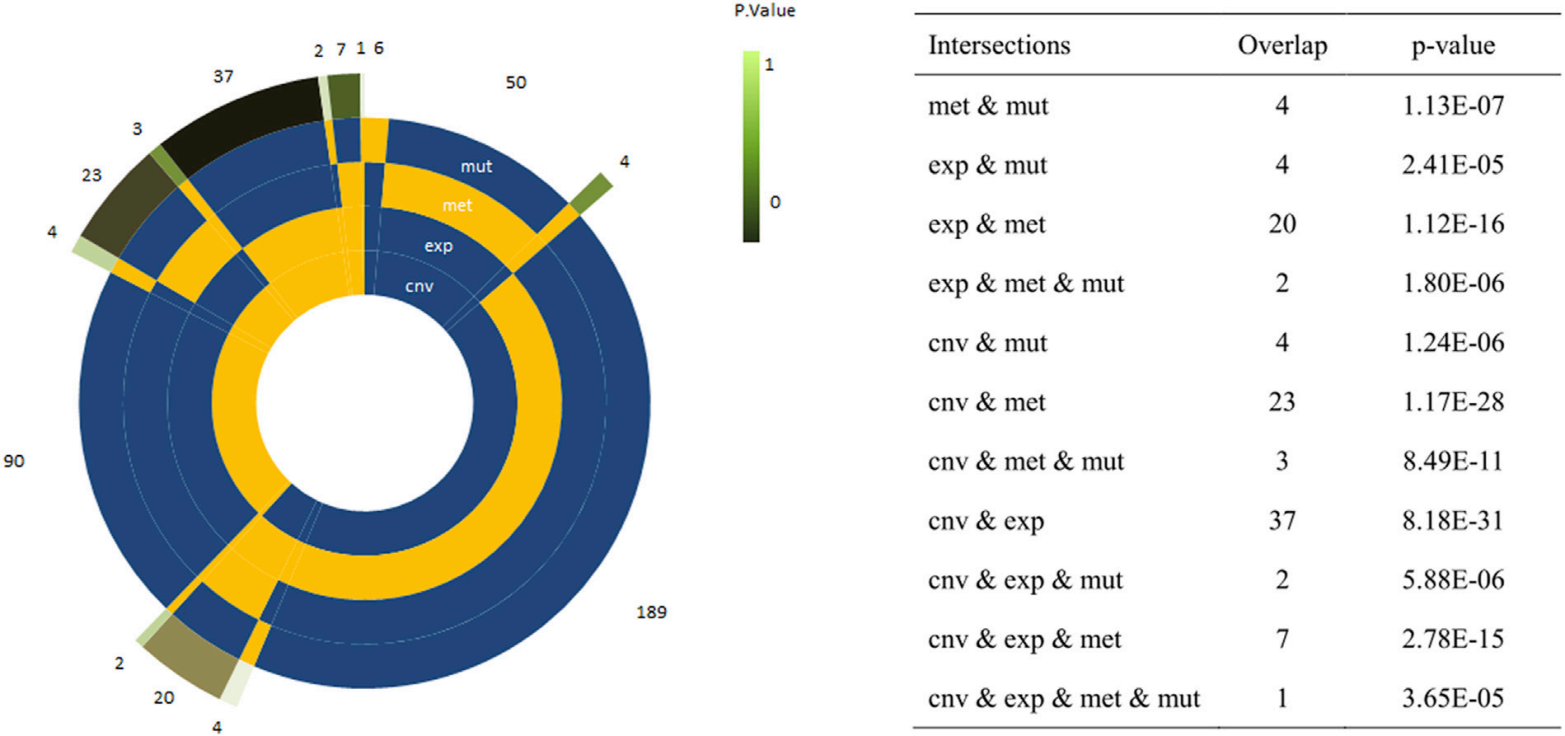
Circle plots representing the frequency of modules with a single or a combination of significant omics variables. The four innermost layers represent the combination of omics (the yellow sector means ‘presence’/the blue sector means ‘absence’), while the external layer represents the frequency of the combination. The explicit *p*-values of the overlap between omic-specific sets of pathway were displayed on the right panel.

A total of 2,185 pathways were downloaded from the Reactome database. Among which, there were 1,317 pathways with more than 10 genes. After resampling, 42 pathways were identified with more than 80% success rates, which were significantly associated with survival (Figure 1). In total, 752 of the 2,185 pathways were used for module analysis. These 752 pathways were divided into 5,190 modules and resampled to obtain 265 modules (involving 111 pathways) with more than 80% resampling success rates, which were remarkably related to survival. Gene expression guides survival association in 189 modules, methylation in 50, mutation in 6, and CNVs in 90 modules (Figure 2). After taking the intersection between 42 and 111 pathways above mentioned, a total of 4 pathways (containing more than two omics) were identified, including integrin cell surface interactions (R-HAS-216083), antigen presentation: folding, assembly and peptide loading of class I MHC (R-HAS-983170), signaling by retinoic acid (R-HSA-5362517), and factors involved in megakaryocyte development and platelet production (R-HSA-983231). In view of related literature report and further study, pathway of integrin cell surface interactions was chosen as the main core of this study.

### Pathway of integrin cell surface interactions

**FIGURE 3:**
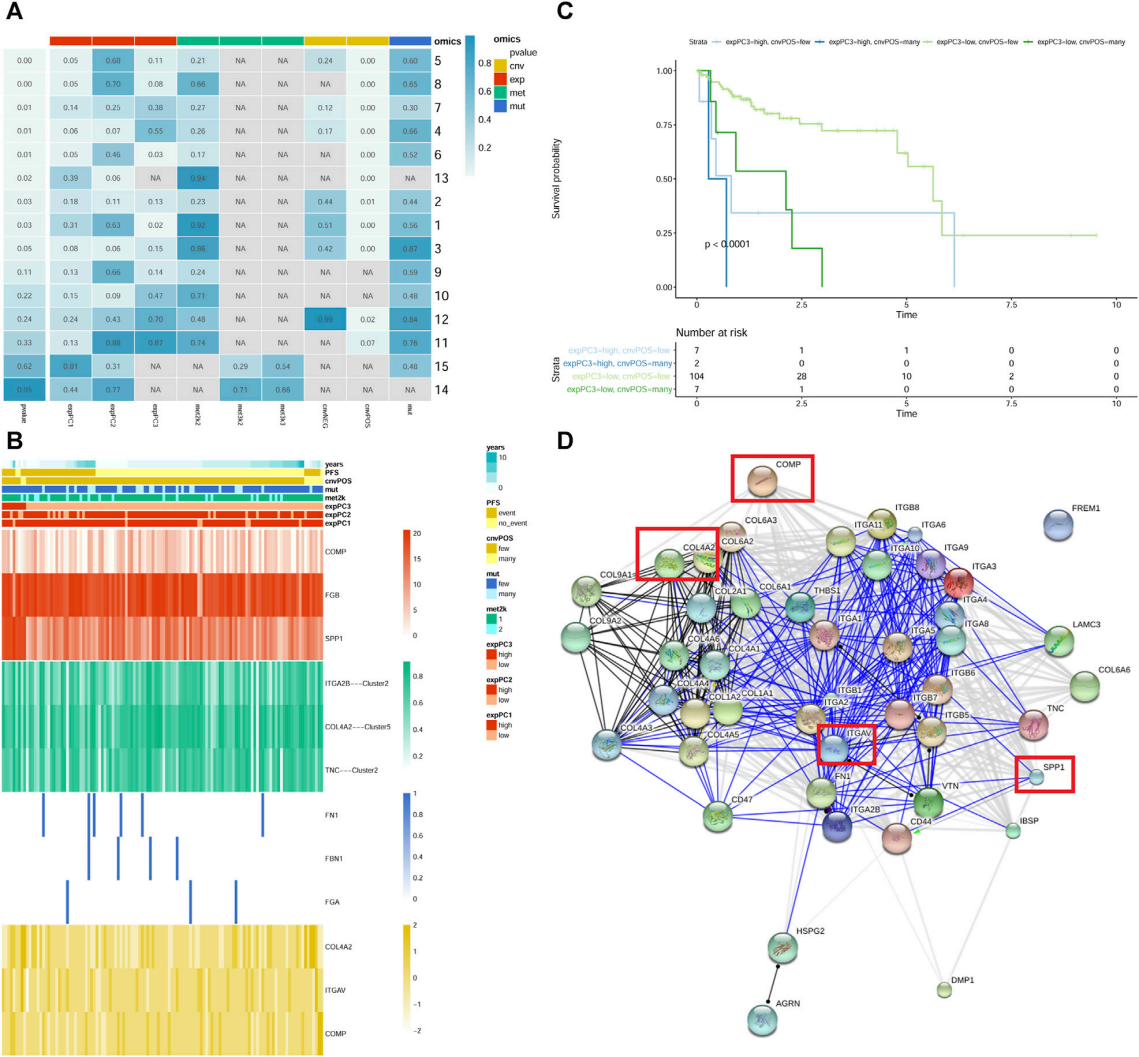
The “integrin cell surface interactions” signaling pathway. **(A)** module summary of the pathway. Row and column represents pathway and omics, respectively. The leftmost *p*-value represents the pathway significance after resampling. *p*-value in the box represents the significance of different omics in the pathway after Cox survival analysis. NA represents no omics in the pathway. cnv: copy number variation data; exp: gene expression data; met: DNA methylation data; mut: mutations data. The cnvPOS and cnvNEG represents positive and negative, respectively. **(B)** heatmap of module 6 of the pathway. The heatmap shows the profiles of prioritized genes for each omics. On top sample annotations are reported. **(C)** The Kaplan–Meier curves of module 6 of the pathway. Patient groups were defined using the combination of expression and CNVs classes. **(D)** PPI network of all genes in the pathway. The genes in the red frame are those screened out.

In pathway of integrin cell surface interactions, the number 6 (Figure 3A) was found, in which gene expression and CNVs well predicted patients’ survival. It is noted that cartilage oligomeric matrix protein (COMP), fibrinogen beta chain (FGB) and secreted phosphoprotein 1 (SPP1) genes were clearly the most representative for expression; COMP, collagen type IV alpha 2 chain (COL4A2) and integrin subunit alpha V (ITGAV) genes were significantly the most representative for CNVs (Figure 3B). Combining the expression and CNVs variables, we found that patients characterized by low expression of COMP, FGB and SPP1, and few level of CNVs (COMP, COL4A2 and ITGAV) had a significantly better prognosis (Figure 3C). The PPI network of all genes in the “integrin cell surface interactions” pathway was shown in Figure 3D.

### Construction of prognostic risk scoring model based on 4 key genes in pathway of integrin cell surface interactions

**FIGURE 4:**
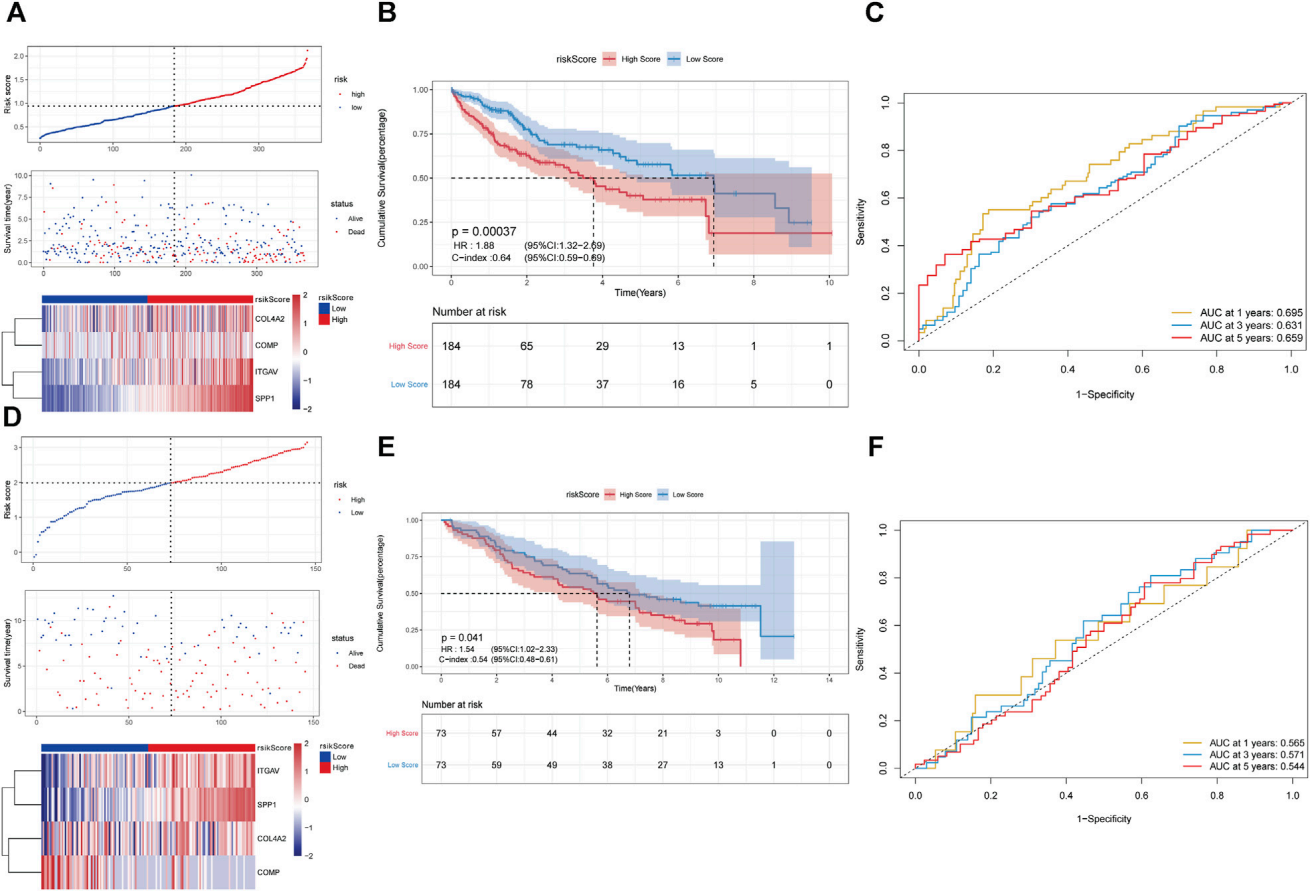
Construction of prognostic risk scoring model based on 4 key genes in pathway of integrin cell surface interactions. **(A–C)**: the analysis of risk score distribution, survival curve and ROC curve in the TCGA database. **(D–F)**: the analysis of risk score distribution, survival curve and ROC curve in the GSE141198 dataset.

In pathway of integrin cell surface interactions, COL4A2, COMP, ITGAV, and SPP1 were used to perform LASSO Cox regression analysis to construct the prediction model of the overall survival rate of liver cancer patients in the TCGA database. The minimum partial likelihood deviation was used to obtain the best λ value. Multivariate Cox regression was used to construct the risk scoring model with the following formula: Risk Score = (COL4A2 * 0.065143343) + (COMP * −0.189426999) + (ITGAV * 0.074265423) + (SPP1 * 0.124743891). According to the median of the risk score, all samples were divided into the high- and low-risk groups. It can be observed that there are a higher proportion of death samples in the high-risk group (Figure 4A). Furthermore, COL4A2, COMP, ITGAV, and SPP1 were upregulated in the high-risk group. Survival analysis indicated that the overall survival of patients in the high-risk group was significantly lower than that in the low-risk group (Figure 4B). The consistency index was 0.64. AUC values of risk score for 1-, 3-, and 5-year survival were 0.695, 0.631 and 0.659, respectively (Figure 4C). The predictive ability of the risk score was validated in GSE141198 dataset. Similarly, a higher proportion of death samples were observed in the high-risk group (Figure 4D). In the high-risk group, the overall survival of patients was significantly lower than that in the low-risk group, with a consistency index of 0.54 (Figure 4E). AUC values of risk score for 1-, 3- and 5-year survival were 0.565, 0.571, and 0.544, respectively (Figure 4F).

### Relationship between risk score and clinical features

**FIGURE 5:**
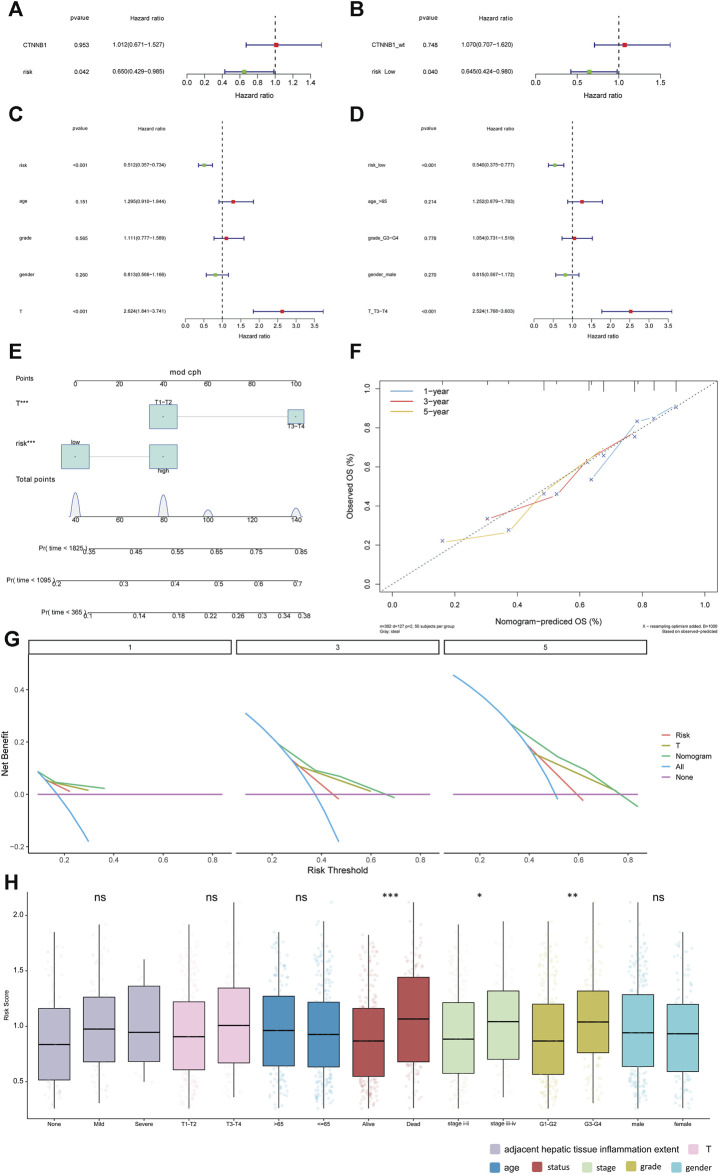
Relationship between risk score and clinical features. **(A, B)**: univariate Cox analysis and multivariate Cox analysis in the TCGA database. **(C, D)**: univariate Cox analysis and multivariate Cox analysis in the GSE141198 dataset. **(E)**: Nomogram of clinical features and risk score. **(F)**: calibration curve of the nomogram in 1-year, 3-year and 5-year. **(G)**: net benefit analysis of nomogram by DCA. Purple line means none of the samples were processed (the net benefit is 0). Blue line indicates that all samples have been processed. The *X*-axis represents the threshold probability experienced by the patient. If the curve is close to two reference (purple and blue) lines, the model has no application value. While if the curve is higher than two reference lines in a large threshold interval, the model is better. **(H)**: differences in risk score among different clinical subgroups. *p < 0.05; **p < 0.01; ***p < 0.001. ns: not significant.

The univariate and multivariate Cox analysis were used to determine the correlation between risk score and clinical features. The result showed that T staging and risk score could be used as independent prognostic factors (Figures 5A,B). The result was validated in the GSE141198 dataset (Figures 5C,D). A nomogram was constructed to predict the 1-, 3- and 5-year overall survival probability of liver cancer patients by combining the risk score with and T stage (Figure 5E). Calibration curves showed that the nomogram had a high accuracy in 1-year overall survival (Figure 5F). These results suggest that, compared with the use of single prognostic factor, the nomogram constructed by multiple factors may be a better predictor of short-term survival in patients with liver cancer. Additionally, the potential net benefit of the prognostic model was further demonstrated through DCA. DCA curves of T stage, risk score and nomogram at 1-, 3- and 5-year were analyzed (Figure 5G). The result showed that nomogram (DCA curve in green) had a higher contribution to predict prognosis at each time point. The differences of risk score in different clinical subgroups were also compared (Figure 5H). The result showed that the risk score was significantly different in the tumor status, stage and grade groups.

### Expression analysis and ceRNA network construction of 4 key genes in the prediction model

**FIGURE 6:**
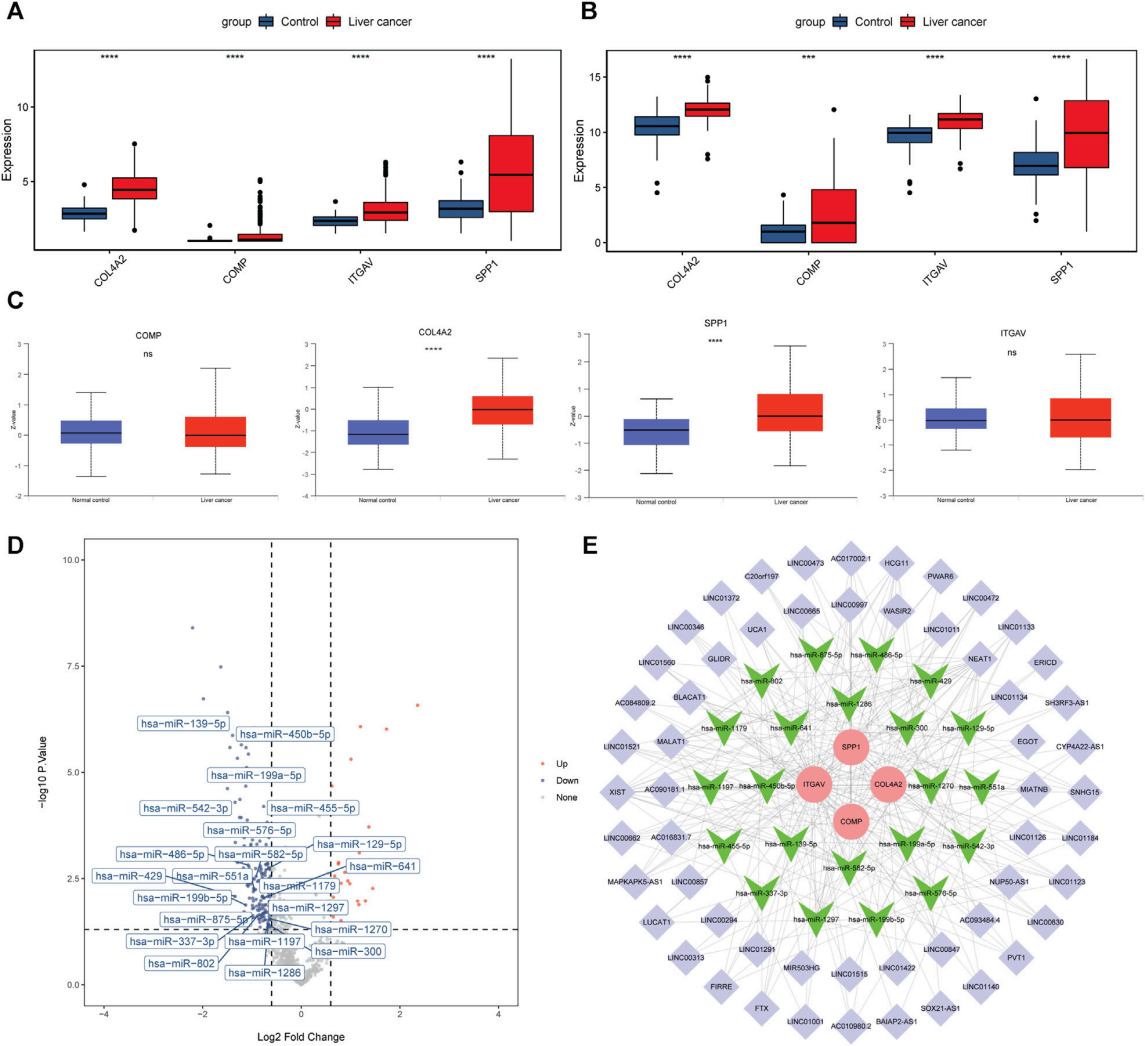
Expression analysis and ceRNA network construction of 4 key genes in the prediction model. **(A, B)**: mRNA expression levels of 4 key genes in the TCGA database and GSE144269 dataset. **(C)**: protein expression levels of 4 key genes in the UALCAN database. ***p < 0.001; ****p < 0.0001. ns: not significant. **(D)**: volcano map of 22 miRNAs in the GSE36915 dataset. **(E)**: ceRNA regulatory network based on 4 key genes, 22 miRNAs and 56 lncRNAs. Round, inverted triangle and purple diamond represent key gene, miRNA and lncRNA, respectively. Red and green represent upregulation and downregulation.

The mRNA expression levels of COL4A2, COMP, ITGAV, and SPP1 were significantly increased in liver cancer tissues (Figure 6A). The result was validated in the GSE144269 dataset (Figure 6B). Protein expressions of COL4A2, COMP, ITGAV, and SPP1 were analyzed based on UALCAN database. COL4A2 and SPP1 were significantly upregulated in liver cancer tissues (Figure 6C). A total of 404 miRNA-mRNA pairs, including 308 miRNAs, were obtained in ENCORI database. In the GSE36915 dataset, a total of 204 differentially expressed miRNAs were identified in liver cancer, including 28 upregulated and 176 downregulated miRNAs. After taking interaction between 308 predicted miRNAs and 176 downregulated miRNA, a total of 22 downregulated miRNAs were obtained (Figure 6D). LncRNAs related to these 22 miRNAs were searched based on the ENCORI database. Totally, 703 lncRNA-miRNA pairs were found, involving 411 lncRNAs. Among which, 162 lncRNAs were differentially expressed in liver cancer in the TCGA database. The correlation between 162 lncRNAs and 4 key genes was analyzed. A total of 56 lncRNAs were positively associated with 4 key genes. The ceRNA regulatory network was constructed based on 4 upregulated key genes, 22 downregulated miRNAs and 56 upregulated lncRNAs (Figure 6E). Some ceRNA pairs were identified, such as NEAT1/BAIAP2-AS1-ITGAV-hsa-miR-542-3p and MAPKAPK5-AS1-ITGAV/SPP1-hsa-miR-450b-5p.

### Analysis of prognosis, diagnosis and drug prediction of 4 key genes in the prediction model

**FIGURE 7:**
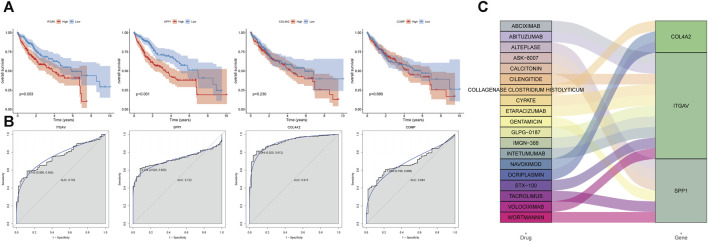
Analysis of prognosis, diagnosis and drug prediction of 4 key genes in the prediction model. **(A)** survival curve. **(B)** ROC curve. **(C)** drug prediction.

Based on the expression levels of 4 key genes, patients were divided into high and low expression groups according to the median expression. Patients with low expression of ITGAV and SPP1 genes had longer overall survival (Figure 7A). COL4A2 and COMP were found to have no significant effect on survival. In addition, ROC analysis showed that these 4 key genes had a potential diagnostic value for liver cancer patients (Figure 7B). The AUC values of COL4A2, ITGAV, SPP1, and COMP were 0.915, 0.75, 0.722, and 0.684, respectively. Finally, based on DGIdb database, 19 drugs were observed to potential target key genes. It is a pity that no drugs are found to be associated with COMP. COL4A2 was targeted by 3 drugs, ITGAV was targeted by 10 drugs, and SPP1 was targeted by 6 drugs (Figure 7C). For instance, ITGAV was targeted by CILENGITIDE. SPP1 was targeted by both WORTMANNIN and TACROLIMUS.

### Association analysis between risk score, 4 key genes and immune cell infiltration

**FIGURE 8:**
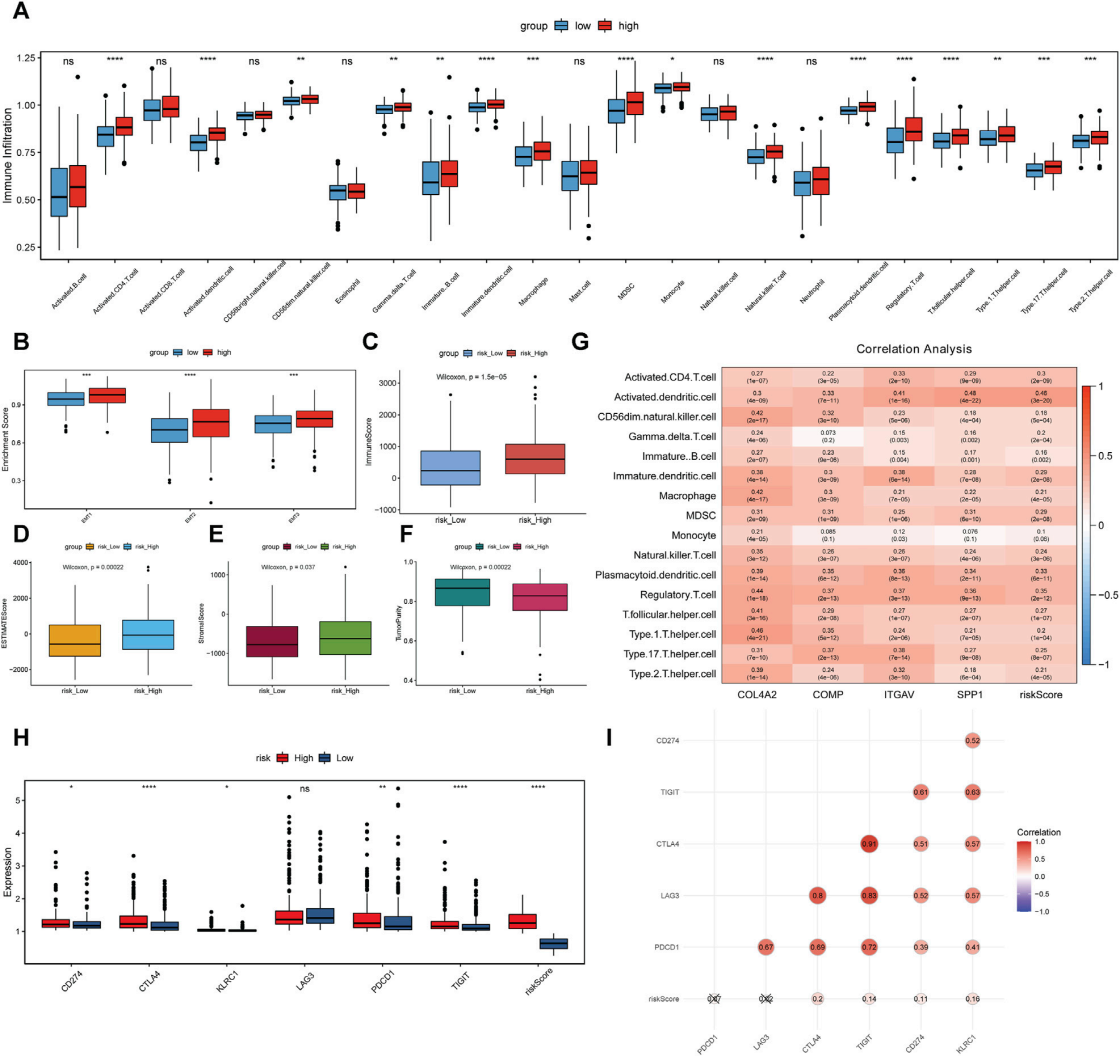
Association analysis between risk score, 4 key genes and immune cell infiltration. **(A–F)**: differences of infiltration degree of 23 kinds of immune cells, EMT, immune score, ESTIMATE score, stromal score and tumor purity in high-risk and low-risk groups. **(G)**: association analysis. **(H)**: differences of immune checkpoints in high-risk and low-risk groups. *p < 0.05; **p < 0.01; ****p < 0.0001. ns: not significant. **(I)**: association between immune checkpoints and risk score.

The immune cell infiltration levels in high-risk and low-risk groups were evaluated using ssGSEA. Infiltration level of 16 kinds of immune cells was significantly higher in the high-risk group than that in the low-risk group (Figure 8A). Moreover, epithelial mesenchymal transition (EMT) 1, EMT2 and EMT3 were significantly higher in the high-risk group, suggesting that matrix activation suppresses the antitumor effect of immune cells (Figure 8B). In addition, the immune score (Figure 8C), ESTIMATE score (Figure 8D), and stromal score (Figure 8E) were significantly higher in the high-risk group. On the other hand, the tumor purity was lower in the high-risk group than that in the low-risk group (Figure 8F). Correlation analysis showed that the risk score and 4 key genes were positively related to 16 kinds of immune cells (Figure 8G). For example, risk score, ITGAV, and SPP1 were the most significantly positively associated with activated dendritic cell. COL4A2 and COMP were the most significantly positively associated with Type 1 T helper cell and regulatory T cell, respectively. In immune checkpoint analysis, cytotoxic T-lymphocyte associated protein 4 (CTLA4) and T cell immunoreceptor with lg and ITIM domains (TIGIT) were the two most significantly upregulated immune checkpoints in the high-risk group compared with low-risk group (Figure 8H). Furthermore, CTLA4 was the most significantly positively associated with risk score (Figure 8I).

### RT-qPCR validation of key genes associated with patient survival

**FIGURE 9:**
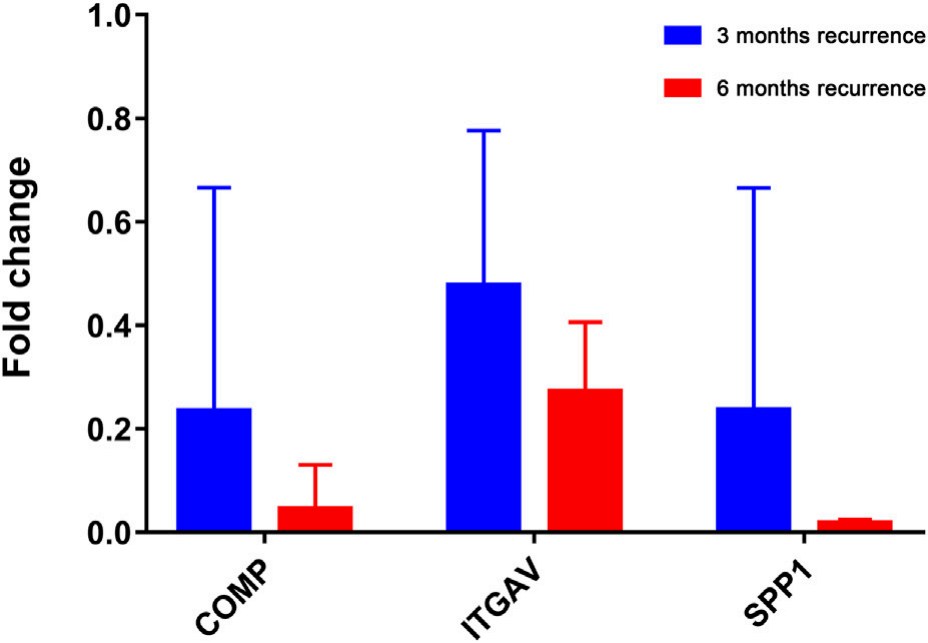
The *in vitro* expression analysis of COMP, ITGAV and SPP1 in patients who relapsed after 3 and 6 months. Fold change >1 and fold change <1 represents upregulation and downregulation, respectively.

The RT-qPCR experiment was applied to further analyze the expression patterns of 3 key genes (COMP, ITGAV, and SPP1) in liver cancer patients who relapsed after 3 and 6 months. Clinical information of these patients was presented in Supplementary Table S1. The expression of these genes was downregulated in patients who relapsed after 6 months compared with that in patients who relapsed after 3 months (Figure 9). Although the result was not significant, the trends were in line with those of previous analysis, probably due to the small sample size and the large heterogeneity among samples. That is to say that low expression of COMP, ITGAV, and SPP1 is associated with better prognosis of patients.

### Overexpression of ITGAV promotes tumor cell proliferation and inhibits tumor cell apoptosis

**FIGURE 10:**
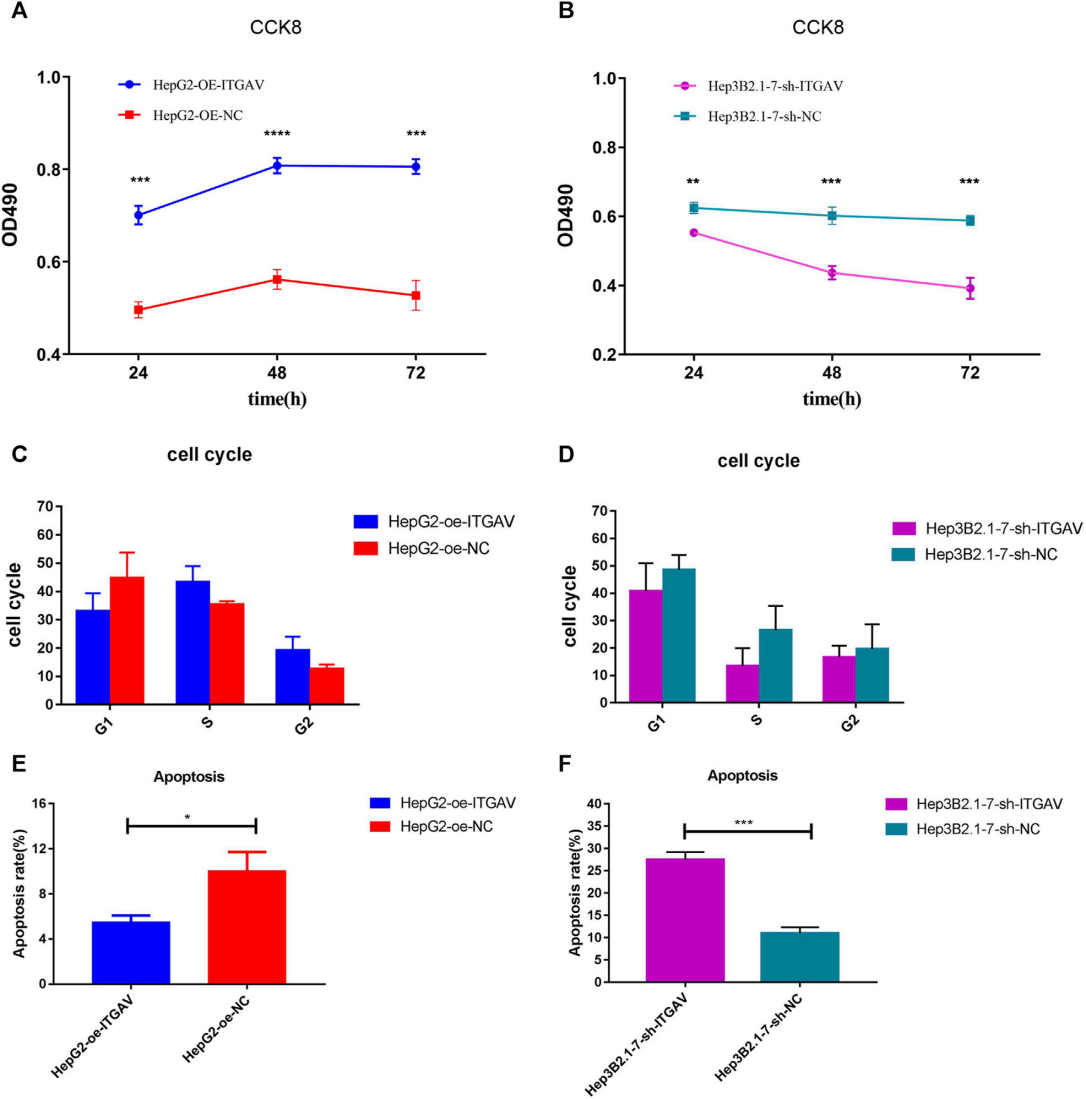
Overexpression of ITGAV promotes tumor cell proliferation and inhibits tumor cell apoptosis. **(A, C and E)**: the effect of over expression of ITGAV on cell proliferation, cell cycle and cell apoptosis; **(B, D and F)**: the effect of knockdown of ITGAV on cell proliferation, cell cycle and cell apoptosis. *p < 0.05; **p < 0.01; ***p < 0.001.

In view of previous analysis results and related literature reports, one of the key genes, ITGAV, was used for further molecular mechanism analysis. From the CCK-8 experiment, we found that overexpression of ITGAV obviously promoted tumor cell proliferation at 48 and 72 h after transfection, while knockdown of ITGAV markedly inhibited the proliferation of tumor cells (Figures 10A,B). However, no effect of ITGAV overexpression/knockdown on the cell cycle was observed (Figures 10C,D). In the cell apoptosis analysis, overexpression of ITGAV significantly inhibited tumor cell apoptosis (Figure 10E). Correspondingly, knockdown of ITGAV remarkably promoted tumor cell apoptosis (Figure 10F).

### Overexpression of ITGAV promotes tumor cell migration and invasion

**FIGURE 11:**
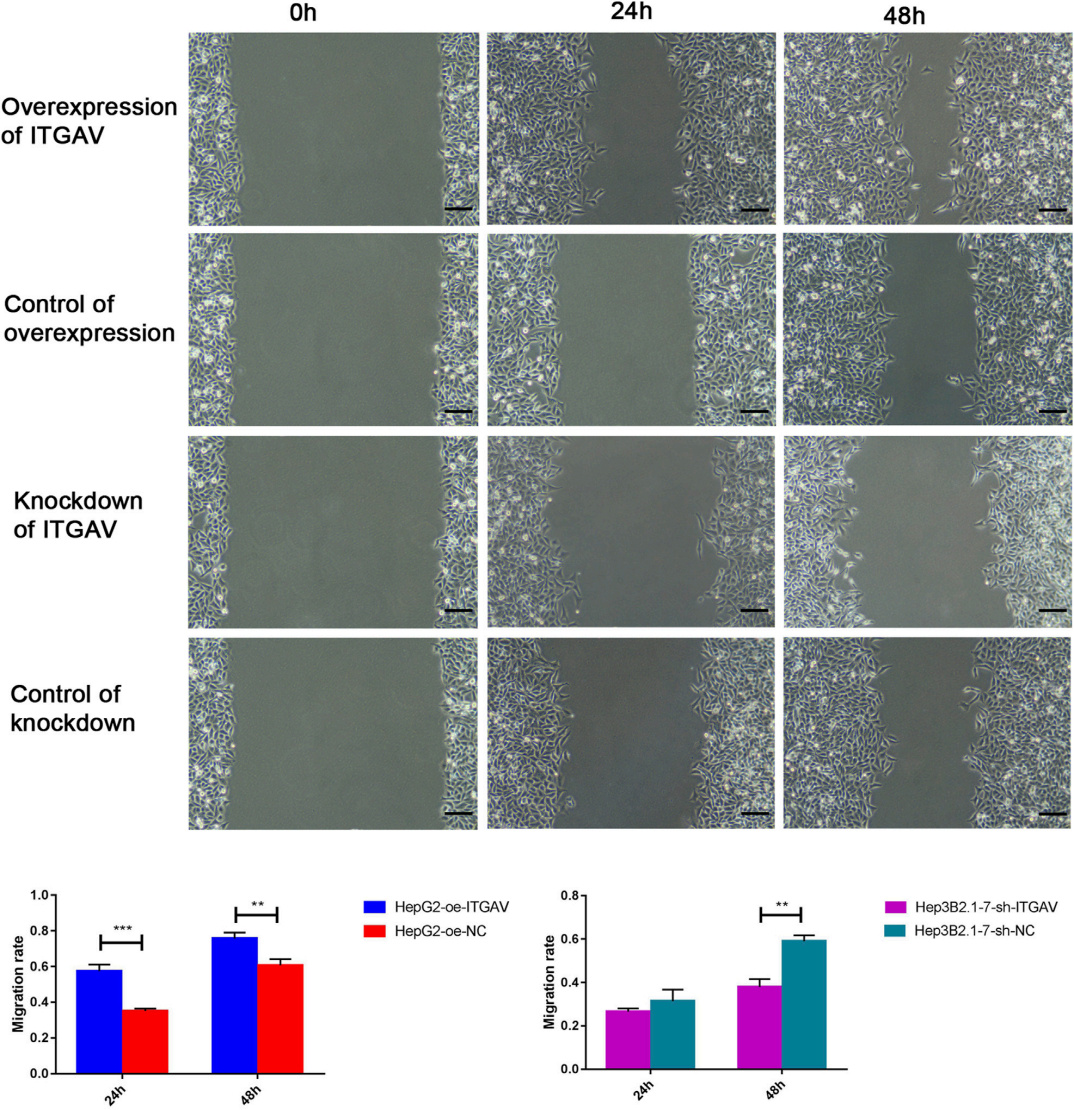
Overexpression of ITGAV promotes tumor cell migration in the cell scratches assay. The experiments were replicated three times. Scale bar = 100 μm **p < 0.01; ***p < 0.001.

**FIGURE 12:**
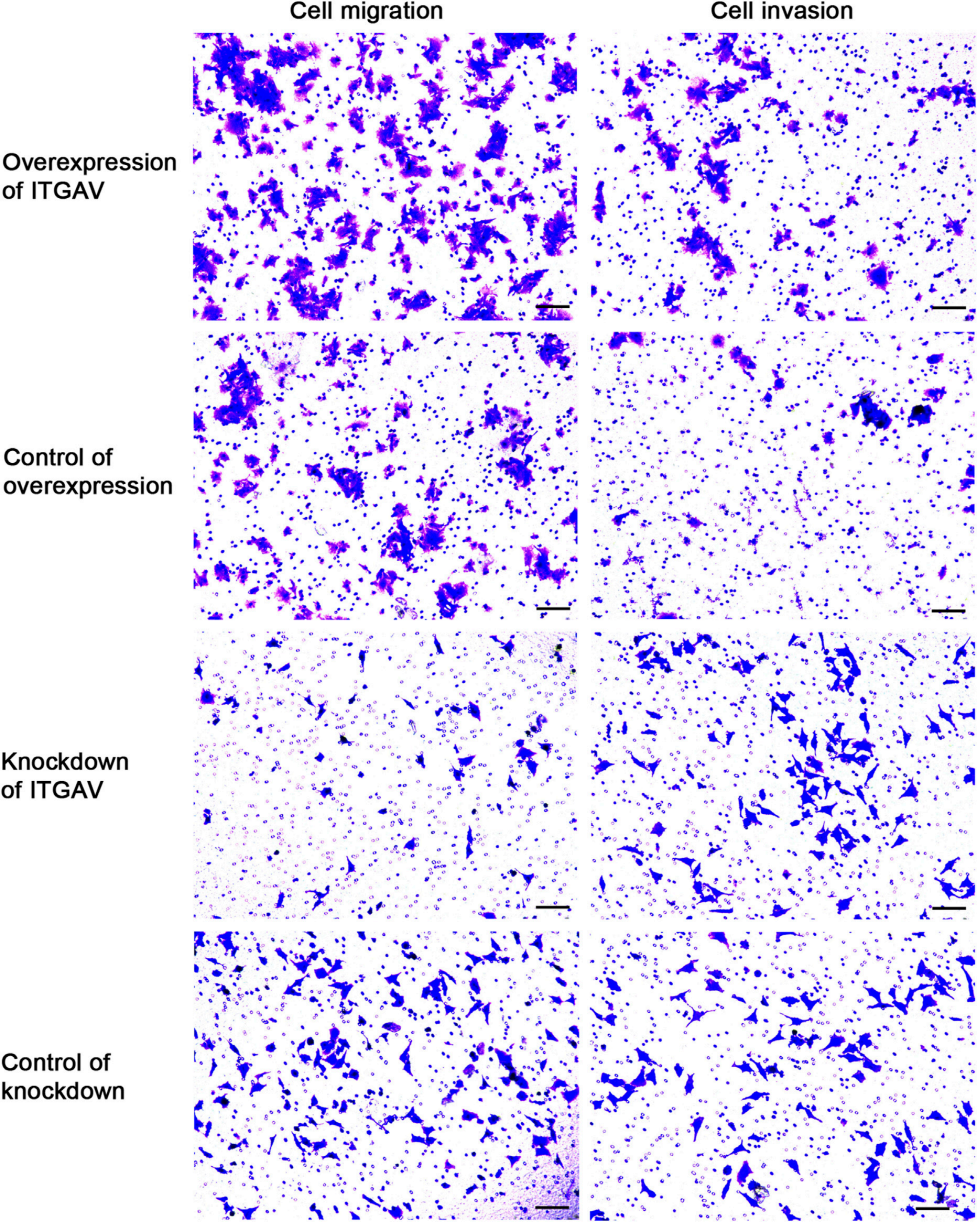
Overexpression of ITGAV promotes tumor cell migration and invasion in the Transwell assay. The experiments were replicated three times. Scale bar = 100 μm.

Cell sratches analysis (Figure 11) showed that overexpression of ITGAV significantly promoted the migration of tumor cells at 24 and 48 h after transfection, while knockdown of ITGAV remarkably inhibited the migration of tumor cells. The result of transwell assay (Figure 12) also showed that overexpression of ITGAV promoted the migration and invasion of tumor cells. Knockdown of ITGAV inhibited the migration and invasion of tumor cells.

### Western blotting analysis

**FIGURE 13:**
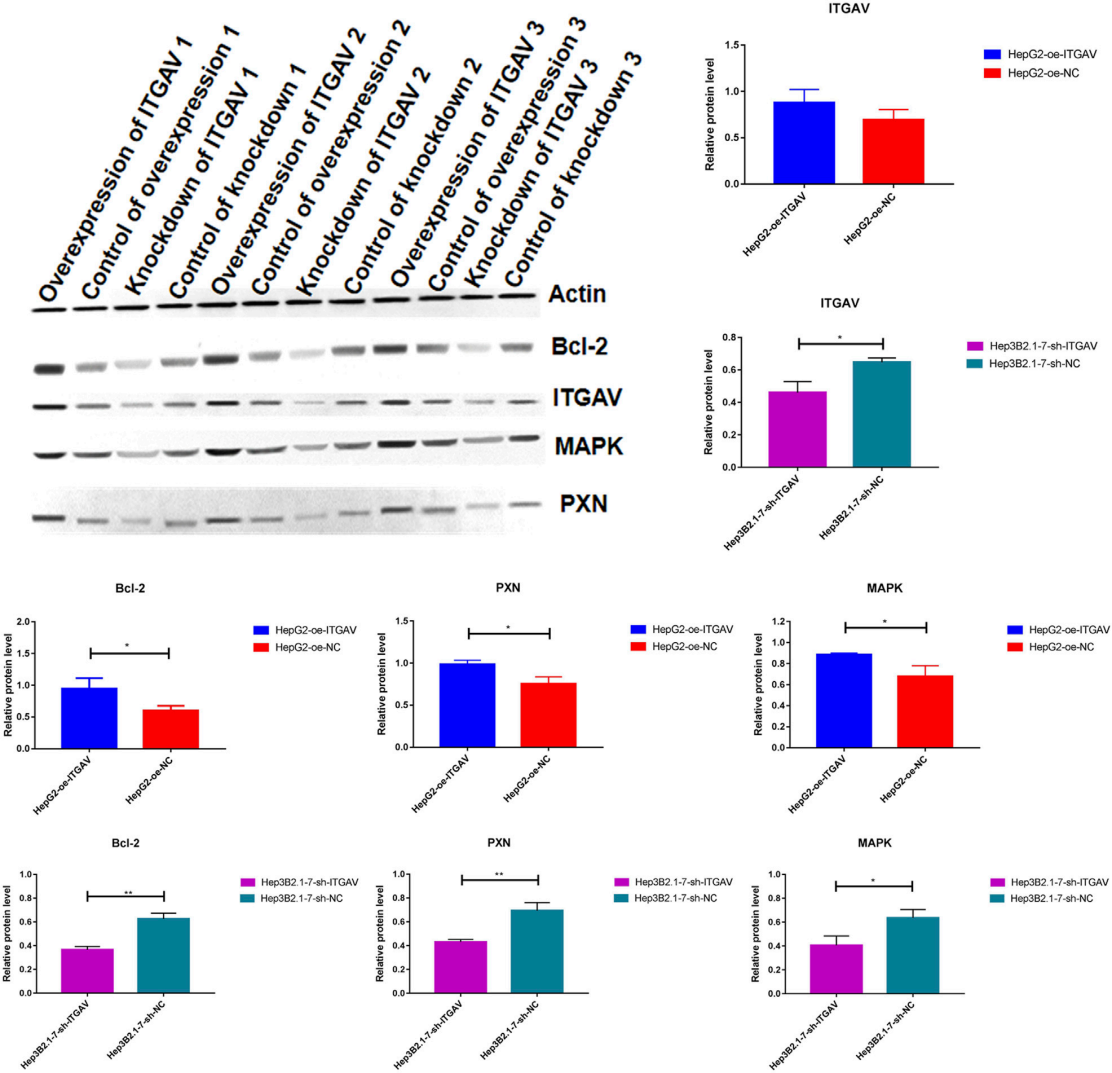
The effect of over expression and knockdown of ITGAV on the protein expression of BCL2, PXN, and MAPK. *p < 0.05; **p < 0.01.

It is reported that ITGAV silencing inhibits cell proliferation, invasion and self-renewal of breast cancer cell lines by altering the expression of BCL2 apoptosis regulator (BCL2) and paxillin (PXN) (Cheuk et al., 2020). In addition, changes in integrin expression activate downstream proteins through cascade phosphorylation of mitogen-activated protein kinase (MAPK). In order to explore the molecular mechanism by which ITGAV promotes tumorigenesis of liver cancer, the protein expression of BCL2, PXN, and MAPK was detected after overexpression and knockdown of ITGAV. The result showed that overexpression of ITGAV significantly increased the expression levels of BCL2, PXN, and MAPK, while knockdown of ITGAV would decrease the expression levels of BCL2, PXN, and MAPK (Figure 13). This indicated that ITGAV may play important roles in tumorigenesis of liver cancer by activating the expression of BCL2, PXN and MAPK.

## Discussion

Integrin plays important roles in the regulation of cell proliferation, differentiation, migration and survival. The expression pattern of integrin is different and upregulated in primary and metastatic liver cancer tissues (Sircana et al., 2019). Within the integrin family, integrin β1 has been studied in solid tumors (Lugano et al., 2018; Zhang et al., 2022). Knockdown of integrin β1 could slow down liver cancer progression by inhibiting MET proto-oncogene, receptor tyrosine kinase (MET) and epidermal growth factor receptor signaling (Bogorad et al., 2014; Speicher et al., 2014). In addition, enhanced integrin signaling by the extracellular matrix and mammalian target of rapamycin (mTOR)/AMP-activated protein kinase (AMPK)-prominin 1 (CD133) is involved in liver tumorigenesis (Chen et al., 2015; Sircana et al., 2019). Interestingly, the expression of integrin is positively associated with poor prognosis in liver cancer (Masumoto et al., 1999). In this study, we found that integrin cell surface interactions signaling pathway was significantly related to survival in liver cancer patients. Based on 4 genes (COMP, SPP1, COL4A2, and ITGAV) in the pathway of integrin cell surface interactions, a risk score model for prognosis of liver cancer was constructed. The median risk score was used as the cut-off point to divide patients into the high- and low-risk groups. In this study, we found that COMP, SPP1, COL4A2, and ITGAV were upregulated in the high-risk group. The overall survival of patients in the high-risk group was significantly lower. Furthermore, the T stage and risk score could be used as independent prognostic factors. The nomogram (based on T stage and risk score) could be a better predictor of short-term survival in patients with liver cancer.

The combination of different omics biomarkers and model construction methods had been applied to increase accuracy of diagnosis and prognosis prediction. Integrative analysis of DNA methylation and gene expression identified several hepatocellular carcinoma (HCC)-specific CpG islands with promising diagnostic value (Cheng et al., 2018). Deep learning-based multi-omics (including mRNA expression, miRNA expression, CpG methylation and clinical information) integration identified two subtypes model of HCC patients with independent predictive values on patient survival in TCGA cohort (Chaudhary et al., 2018). Herein, we applied MOSClip for the first time to integrate multi-omics data (including gene expression, mutations, CNVs, and DNA methylation data) of liver cancer to construct a prognostic model for predicting prognosis. The risk scoring model constructed by four genes achieved good prognosis prediction effect and was validated in the validation dataset. Compared with previous studies, this study adopted a new analysis method and covered more omics data, which provided a more comprehensive perspective and deeper analysis for HCC prognosis research.

It has been proposed that COMP is a new non-invasive biomarker to assess the risk of liver cancer (Andréasson et al., 2015; Norman et al., 2015). It is found that liver cancer-associated fibroblasts secrete COMP to promote proliferation, invasion, migration and EMT in liver cancer cells (Sun et al., 2019). SPP1 is associated with liver cancer risk in cirrhosis (Mazziotti et al., 2002; Duarte-Salles et al., 2016). SPP1 has been regarded as a preclinical target for liver cancer treatment (Zheng et al., 2020). The model of SPP1 combined with centromere protein A (CENPA), melanoma-associated antigen family member B6 (MAGEB6), and homeobox D9 (HOXD9) can predict the overall survival in liver cancer patients (Long et al., 2018). Additionally, models consisting of SPP1 and lecithin-cholesterol acyltransferase (LCAT) are good at predicting liver cancer diagnosis, prognosis and recurrence (Long et al., 2019). COL4A2 is a significantly upregulated gene in liver cancer cells (Wang et al., 2020). Splice variants for COL4A2 are co-up-regulated in the liver cancer tumors (Lai et al., 2011). Over expression of COL4A2 is highly related to shorter progression-free survival in patients with liver cancer (Liu et al., 2020). Thus, it can be seen that COMP, SPP1, and COL4A2 play important roles in the development of liver cancer.

Overexpressed ITGAV is found in liver cancer (Xia et al., 2014). It is worth mentioning that ITGAV is a potential immune related prognostic index for liver cancer patients (Zhang et al., 2020). Herein, we found that ITGAV was regulated by some lncRNAs and miRNAs. Some ceRNA relational pairs were identified, including NEAT1/BAIAP2-AS1-ITGAV-hsa-miR-542-3p and MAPKAPK5-AS1-ITGAV-hsa-miR-450b-5p. NEAT1 is specifically overexpressed in liver cancer (Fujimoto et al., 2016). NEAT1 overexpression is an independent risk factor related to the prognosis of liver cancer patients (Liu et al., 2017). Increased expression levels of BAIAP2-AS1 are found in patients with liver cancer (Yang et al., 2021). Moreover, knockdown of BAIAP2-AS1 inhibited the proliferation and metastasis of liver cancer cells. MAPKAPK5-AS1 expression is elevated in liver cancer (Wang et al., 2021). Furthermore, high expression of MAPKAPK5-AS1 is associated with advanced stage and lymph node metastasis of patients with liver cancer. Hsa-miRNA-542-3p, downregulated in liver cancer, inhibits tumor cell growth (Wu et al., 2017). Hsa-miR-450b-5p loss promotes liver cancer progression (Li et al., 2019). It is found that hsa-miR-450b-5p is negatively related to survival in liver cancer patients (Pascut et al., 2020). It is suggested that NEAT1/BAIAP2-AS1-ITGAV-hsa-miR-542-3p and MAPKAPK5-AS1-ITGAV-hsa-miR-450b-5p axis may be involved in the process of liver cancer. The results of cell assay showed that overexpression of ITGAV promoted tumorigenesis. Furthermore, overexpression of ITGAV significantly increased the expression levels of BCL2, PXN, and MAPK. It is reported that ITGAV silencing inhibits cell proliferation, invasion, and self-renewal of breast cancer cell lines by altering the expression of BCL2 and PXN (Cheuk, Siu, 2020). In addition, changes in integrin expression activate downstream proteins through cascade phosphorylation of MAPK. It is suggested that ITGAV may play important roles in tumorigenesis of liver cancer by activating the expression of BCL2, PXN, and MAPK.

It is well known that the immune response plays important roles in the development of tumors. In this study, the infiltration level of 16 kinds of immune cells was significantly higher in the high-risk group compared with the low-risk group. The immune score, ESTIMATE score and stromal score were significantly higher in the high-risk group than those in the low-risk group. Moreover, EMT1, EMT2 and EMT3 were significantly higher in the high-risk group. EMT endows epithelial cells with invasive and migratory capacity in metastases of liver cancer (Thiery et al., 2009; Jou and Diehl, 2010). In immune checkpoint analysis, CTLA4 and TIGIT were the two most significantly upregulated immune checkpoints in the high-risk group. TIGIT expression promotes liver cancer progression through tumor-associated immune suppression (Zheng et al., 2020). It is suggested that matrix activation and expression of immune checkpoints are related to the antitumor effect of immune cells in the high-risk group. Correlation analysis showed that ITGAV and SPP1 were the most significantly positively associated with activated dendritic cell. COL4A2 and COMP were the most significantly positively associated with Type 1 T helper cell and regulatory T cell, respectively. Mature dendritic cells can mediate liver cancer immune evasion (Lurje and Hammerich, 2020). Type 1 T helper cells play important roles in the development of primary liver cancer (Chen et al., 2021). Type 17 T helper cells to type 1 T helper cells ratio is served as a potential prognostic marker for scoring the severity of liver cancer (Yan et al., 2014). In peripheral blood and tumor tissue of liver cancer patients, increased regulatory T cell is associated with tumor stage and patients survival (Beyer and Schultze, 2006; Fu et al., 2007; Gao et al., 2007; Shen et al., 2010; Takata et al., 2011). It is indicated that the association between ITGAV, SPP1, COL4A2, COMP, and immune cells may be involved in the progression of liver cancer.

In addition, ROC analysis showed that ITGAV, SPP1, COL4A2, and COMP had a potential diagnostic value for liver cancer patients. The AUC values of COL4A2, ITGAV, SPP1, and COMP were 0.915, 0.75, 0.722, and 0.684, respectively. Based on DGIdb database, we found that ITGAV was targeted by CILENGITIDE. SPP1 was targeted by both WORTMANNIN and TACROLIMUS. CILENGITIDE, an inhibitor of integrin αvβ3, can suppress tumor progression in clinical trials and improve the outcome in patients (Stupp et al., 2014). The combination of WORTMANNIN and SORAFENIB enhances the inhibitory of liver cancer cell lines (Liu et al., 2022). In patients with liver cancer, high TACROLIMUS intra-patient variability (IPV) is significantly related to an increased risk of overall mortality and disease recurrence (Kim et al., 2022). It is suggested that ITGAV and SPP1 may be considered as potential diagnostic biomarkers and therapeutic targets for liver cancer.

The risk score model constructed by genes (COMP, SPP1, COL4A2, and ITGAV) in the pathway of integrin cell surface interactions may be used to predict survival in liver cancer patients. Our result could be helpful in improving patient outcomes and contributing to diagnosis and treatment decisions for liver cancer. However, there are limitations in our study. Firstly, deeper molecular mechanism of identified genes is not investigated. Some animal models are further needed to study the biological function of identified genes in the disease. Secondly, the molecular profile of identified genes with different samples, such as patients in different stage/grade of liver cancer, is needed in the further study. Thirdly, correlation analysis between clinical information of patients and identified genes is further needed. Last but not least, the sample size used for RT-qPCR validation was too small, resulting in the lack of significance of the results. Validation with a larger sample size should be done in future work.

## Data Availability

The original contributions presented in the study are included in the article/Supplementary Material, further inquiries can be directed to the corresponding authors.

## Abbreviations

AMPK AMP-activated protein kinase; AUC area under the curve; BCL2 BCL2 apoptosis regulator; COMP cartilage oligomeric matrix protein; CENPA centromere protein A; COL4A2 COMP, collagen type IV alpha 2 chain; CNVs copy number variations; CTLA4 cytotoxic T-lymphocyte associated protein 4; DCA decision curve analysis; DNAH17 dynein axonemal heavy chain 17; EMT epithelial mesenchymal transformation; FGB fibrinogen beta chain; FC fold change; GEO Gene Expression Omnibus; GEP granulin precursor; HOXD9 homeobox D9; ITGAV integrin subunit alpha V; Lasso Least absolute shrinkage and selection operator; lncRNAs long non-coding RNAs; MAGEB6 MAGE family member B6; MET MET proto-oncogene, receptor tyrosine kinase; miRNAs micro RNAs; MAPK mitogen-activated protein kinase; PXN paxillin; PCR polymerase chain reaction; CD133 prominin 1; mTOR rapamycin kinase; ROC Receiver Operating characteristic; SPP1 secreted phosphoprotein 1; ssGSEA single Sample gene Set Enrichment analysis; TIGIT T cell immunoreceptor with lg and ITIM domains; TP53 tumor protein p53.
